# Anticoagulant effects of rivaroxaban, prednisone, alone and in combination, in healthy dogs

**DOI:** 10.1111/jvim.16572

**Published:** 2022-11-18

**Authors:** Paige M. Hafner, Andrew J. Mackin, Robert W. Wills, Marjory B. Brooks, John M. Thomason

**Affiliations:** ^1^ Department of Clinical Sciences College of Veterinary Medicine, Mississippi State University Mississippi State Mississippi USA; ^2^ Department of Comparative Biomedical Sciences College of Veterinary Medicine, Mississippi State University Mississippi State Mississippi USA; ^3^ Department of Population Medicine and Diagnostic Sciences College of Veterinary Medicine, Cornell University Ithaca New York USA

**Keywords:** corticosteroid, glucocorticoid, immune‐mediated hemolytic anemia, thromboprophylaxis

## Abstract

**Background:**

The potential effects of glucocorticoid administration on rivaroxaban's anticoagulant bioactivity in dogs, and an appropriate rivaroxaban dosage regimen for dogs receiving glucocorticoid therapy are unknown.

**Hypothesis/Objectives:**

The objective was to determine whether glucocorticoid administration influences the anticoagulant effects of rivaroxaban in healthy dogs. We hypothesized that administration of rivaroxaban and prednisone would reduce the anticoagulant intensity compared with rivaroxaban alone.

**Animals:**

Nine healthy dogs.

**Methods:**

Randomized, cross‐over study. Dogs were administered prednisone (2 mg/kg, PO, q24h), rivaroxaban (1.5 mg/kg, PO, q24h), or prednisone and rivaroxaban, and the coagulation status was evaluated using prothrombin time (PT), and rivaroxaban‐calibrated anti‐Xa activity (RIVA, results were log_10_ transformed for analysis), before drug administration and on days 2, 4, and 8. Linear mixed models and correlation were used to evaluate associations in variables (*P* < .05 was considered significant).

**Results:**

There were no differences in RIVA results for the rivaroxaban and prednisone/rivaroxaban groups on day 8 (*P* = .599, median 87 [range 45‐156] to 167 [56‐333], respectively, median difference 90 ng/mL [95% CI:87.3‐161.8]) There was a strong correlation between RIVA and PT results when days 2, 4, and 8 were combined (*r* = .846, *P* < .001), and increased during drug administration, day 2 (*r* = .810, *P* < .001), day 4 (*r* = .863, *P* < .001), and day 8 (*r* = .885, *P* < .001).

**Conclusions and Clinical Importance:**

Clotting times in the PT correlate with rivaroxaban levels and may prove useful for drug monitoring. Prednisone administration had no apparent influence on the anticoagulant effects of rivaroxaban in healthy dogs, suggesting that combined therapy will not require dosage adjustments.

**Abbreviations**
ACT: activated clotting time
aPTT: activated partial thromboplastin time
CR: clot rate
FXa: coagulation factor X
IMHA: immune‐mediated hemolytic anemia
PT: prothrombin time
RIVA: rivaroxaban‐specific anti‐Xa activity

## 1 INTRODUCTION

Thromboembolism is a common cause of death in dogs with immune‐mediated hemolytic anemia (IMHA). Glucocorticoids are the cornerstone of therapy for dogs with IMHA, but can cause hypercoagulability in healthy dogs,^1^, ^2^, ^3^ and are a risk factor for thromboembolism in dog with clinical disease.^2^, ^4^, ^5^ Anti‐platelet and anticoagulant medications, used separtely or in combination, are routinely administered as thromboprophylactic agents to dogs with IMHA that are suspected to be hypercoagulable.^6^ With the addition of anticoagulant^7^ or anti‐platelet^8^ agents in dogs with IMHA, survival rates have improved, but dogs continue to develop fatal thromboembolic complications.^7^, ^8^

According to the ACVIM consensus statement on the treatment of IMHA in dogs, anticoagulant medications are considered to be the preferred thromboprophylactic agents.^6^ Rivaroxaban, an oral anticoagulant, has been used in humans to prevent the formation of postoperative deep vein thrombosis and stroke by specifically inhibiting activated coagulation factor X (FXa).^9^, ^10^ In dogs with IMHA, the administration of rivaroxaban is well tolerated, and incidences of hemorrhage and thrombosis are similar between dogs treated with rivaroxaban and dogs treated with clopidogrel and low‐dose aspirin.^11^ Dogs with IMHA are usuallly treated with glucocorticoids; however, it is unknown how concurrent rivaroxaban and glucocorticoid therapy might influence rivaroxaban's anticoagulant effect.

The objective of this study was to determine whether glucocorticoids influence the anticoagulant effects of rivaroxaban in healthy dogs. Our hypothesis was that concurrent administration of rivaroxaban and prednisone would result in reduced anticoagulant intensity compared with rivaroxaban alone.

## 2 METHODS AND MATERIALS

### 2.1 Animals

Nine healthy adult research dogs, 6 intact females and 3 castrated males, were used in this study. The median age of the dogs was 6.4 years (range, 4.5‐7 years) and the median weight was 23.7 kg (range, 22.2‐40.5 kg). The dogs were deemed healthy based on no abnormalities on physical examination, complete blood count (including manual platelet count), serum biochemistry, urinalysis, and heartworm and tick‐borne disease testing. The dogs were not vaccinated or administered any medications for at least 2 weeks prior to the initiation of the study. A sample size calculation was performed based on the mean and SD of prothrombin time (PT) values when dogs received prednisone^12^ or rivaroxaban^13^ in previous studies. With a power of 95% and a significance level of .05, 8 dogs per treatment group were calculated to be sufficient to identify a 30% increase in PT values during drug administration. Animal use was approved by the XX Institutional Animal Care and Use Committee and was in compliance with the requirements of the American Association of Accreditation of Laboratory Animal Care.

### 2.2 Study design and sample collection

In a 3‐way, randomized, cross‐over study, dogs were given either prednisone (2.0 ± 0.1 mg/kg, [mean ± SD], PO, q24h; Prednisone, West‐Ward Pharmaceutical Corp, Eatontown, New Jersey), rivaroxaban (1.5 ± 0.1 mg/kg, PO, q24h; Janssen Pharmaceuticals, Inc, Titusville, New Jersey), or prednisone (2.0 ± 0.1 mg/kg, PO, q24h) and rivaroxaban (1.5 ± 0.1 mg/kg, PO, q24h). All medications were administered for 8 days, followed by a recovery period for a minimum of 21 days between dosing. After the recovery period, dogs switched groups, and the study was repeated until all dogs had received all medications.

Blood samples were collected before drug administration (day 0) for the following hemostasis tests: activated partial thromboplastin time (aPTT), PT, antithrombin activity, fibrinogen concentration, rivaroxaban‐calibrated anti‐Xa activity (RIVA), and viscoelastometry. On days 2, 4, and 8 of drug administration, approximately 2 hours after morning medication, blood was collected for the following: PT, RIVA, and viscoelastometry. Blood was collected by jugular venipuncture with a 20‐gauge needle directly into 4.5 mL tubes containing 3.2% sodium citrate anticoagulant (3.2% sodium citrate, Vacutainer tube, Becton Dickinson, Franklin Lakes, New Jersey). There were no notable technical problems encountered with venipunctures that could be expected to influence results.

### 2.3 Hemostasis testing

For the measurement of aPTT, PT, antithrombin activity, fibrinogen concentration, and RIVA, citrated blood was centrifuged, and the plasma supernatant was removed and frozen at −80°C. Frozen plasma samples were shipped overnight on dry ice to XX for analysis. All samples were stored at −80°C, until thawed at 37°C immediately before assay. Hemostasis testing was performed within 3 months of collection.

The RIVA assay utilized a commercially available chromogenic substrate assay (STA‐Liquid anti‐Xa, Diagnostica Stago, Parsippany, New Jersey) and the manufacturer's coagulation instrument (STACompact, Diagnostica Stago). The assay reagents consist of bovine FXa and a chromogenic factor Xa substrate. This assay has been previously described for use in pharmacokinetic studies of rivaroxaban in dogs and cats.^14^, ^15^, ^16^ In this assay, rivaroxaban in the test plasma inhibits the bovine FXa reagent. Color change in the assay mixture results from cleavage of the FXa substrate by residual uninhibited bovine FXa and is inversely proportional to the concentration of rivaroxaban in the test plasma. The assay is calibrated with a human plasma standard containing rivaroxaban concentrations defined by mass spectrometry. Assay results are expressed as ng/mL rivaroxaban.

An automated, colorimetric and mechanical endpoint clot detection instrument (StaCompact, Diagnostica Stago) and commercially available coagulation reagents were used to measure aPTT (Dade Actin Fs, Siemens), PT (Thromboplastin LI, Helena Diagnostics), antithrombin activity, and fibrinogen. For measurement of antithrombin activity, a chromogenic substrate kit (Stachrom AT III, Diagnostica Stago) was used according to the manufacturer's recommendations, modified using pooled normal canine plasma, instead of human plasma, as calibration standard. The antithrombin activity was reported as the percentage of the canine standard (assigned value of 100% activity). Plasma concentration of clottable fibrinogen was measured using the Clauss method and a 100 NIH U/mL human thrombin reagent (Fibrinogen, Diagnostica Stago).^17^, ^18^

### 2.4 Viscoelastometry

A viscoelastic coagulation analyzer (SonoClot, Sienco, Inc, Arvada, Colorado) was used to assess coagulation, fibrin gel formation, and clot retraction by detecting mechanical changes within whole blood. Following collection, whole blood samples were held at room temperature for at least 30 minutes,^19^ and analysis was performed based on the manufacturer's recommendations (Operator's Manual, Sienco Inc, Arvada, Colorado). Clotting was triggered using an agonist (gbACT Kit, Sienco Inc, Arvada, Colorado) and the results were calculated with instrument specific software (Signature Viewer Program, Sienco Inc, Arvada, Colorado). Briefly, 330 μL of citrated whole blood and 15 μL 0.2% CaCl_2_ were added to a cuvette, and an oscillating tubular probe was placed in the sample. The probe detects resistance to motion during clot formation and generates a clot signal. The clot signal includes the activated clotting time (ACT; the time until the first detectable clot) in seconds and the clot rate (CR; the slope of the SonoClot signature during the fibrin gel formation) in clot signal units per minute. Samples were analyzed in duplicate and averaged. Quality control analysis was performed at the beginning of each day that samples were analyzed.

### 2.5 Statistical analysis

The effect of treatment on the hemostatic testing and viscoelastometric results were assessed by separate linear mixed models using commercially available statistic software (SAS for Windows v. 9.4, SAS Institute, Inc, Cary, North Carolina). Treatment, run, sample time, and the interaction of treatment and sample time were included as fixed effects. Dog identity was included as a random effect. Repeated measures of dog identity within run for the different sample times were specified in a repeated statement with a spatial power law (SP[POW]) covariance structure to accomodate uneven timing or spacing of measurements. Differences in least squares means, using a simulation‐based adjustment method for multiple comparisons, were used to make pairwise comparisons between treatments within each sample time and between sample times within each treatment for significant interaction terms. To ensure the washout period was sufficient and no carryover effects had occurred, the effect of treatment, run, and their interaction on the day 0 values of each outcome were assessed. The distribution of the conditional residuals for each outcome was assessed to ensure the assumptions of normality and homoscedasticity for the statistical model had been met. The assumptions were not met for RIVA so this outcome was log_10_ transformed for all analyses. The correlation of log_10_ RIVA with ACT, Clot Rate and PT and the correlation of PT with ACT and Clot Rate were determined for sample times day 2, day 4, and day 8. An alpha level of .05 was used to determine statistical significance for all methods.

## 3 RESULTS

### 3.1 Hemostasis testing

The means with SD for the aPTT, fibrinogen concentration, and antithrombin activity before treatment are presented in Table 1. There were no significant differences in these variables, or in PT and RIVA values at the before treatment sample times among the treatment groups.

**TABLE 1:** Before treatment values (mean ± SD) of hemostatic indices from 9 healthy dogs treated with prednisone, rivaroxaban, and prednisone/rivaroxaban for 8 days

	Prednisone	Rivaroxaban	Prednisone/rivaroxaban
aPTT (s)	14.5 ± 1.6	14.8 ± 1.2	14.6 ± 1.5
Fibrinogen (mg/dL)	237.1 ± 58	229.2 ± 60.3	226.8 ± 77.9
Antithrombin activity (%)	89.3 ± 9.6	87.7 ± 11.4	90.3 ± 11.4

**FIGURE 1:**
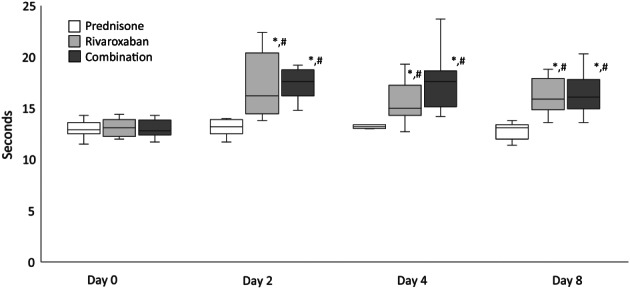
PT values from 9 dogs treated with prednisone, rivaroxaban, and prednisone/rivaroxaban for 8 days. The box and whiskers plot demonstrate the median (line), interquartile range (box), and total range (whiskers). *Indicates a significant (*P* < .05) difference from before treatment (day 0) within a treatment group; ^#^Indicates a significant (*P* < .05) difference from the prednisone group on the corresponding day

The PT results for days 0, 2, 4, and 8 are presented in Figure 1. In the rivaroxaban group, there were significant increases in PT from day 0 to 2 (*P* < .001, median 13.1 [range 12‐14.4] to 16.2 [13.8‐22.4], median difference 3.1 seconds [95% CI: 13.6‐16.7]), day 0 to 4 (*P* = .003, 13.1 [12‐14.4] to 15 [12.7‐19.3] median difference 1.9 seconds [95% CI: 13.4‐15.3]), and day 0 to 8 (*P* < .001, 13.1 [12‐14.4] to 15.9 [13.6‐18.8] median difference 2.8 seconds [95% CI: 13.6‐15.7]). In the rivaroxaban group, there was a significant decrease in PT from day 2 to 4 (*P* = .04, 16.2 [13.8‐22.4] to 15 [12.7‐19.3] median difference 1.2 seconds [95% CI: 15.1‐17.8]).

In the prednisone/rivaroxaban group, there were significant increases in PT from day 0 to 2 (*P* < .001, 12.8 [11.7‐14.3] to 17.6 [14.8‐19.2] median difference 4.8 seconds [95% CI: 14‐16.5]), day 0 to 4 (*p* < .001, 12.8 [11.7‐14.3] to 17.6 [14.2‐23.7] median difference 4.8 seconds [95% CI: 13.7‐16.7]), and day 0 to 8 (*p* < .001, 12.8 [11.7‐14.3] to 16.1 [13.6‐20.3] median difference 3.3 [95% CI: 13.6‐16]).

**FIGURE 2:**
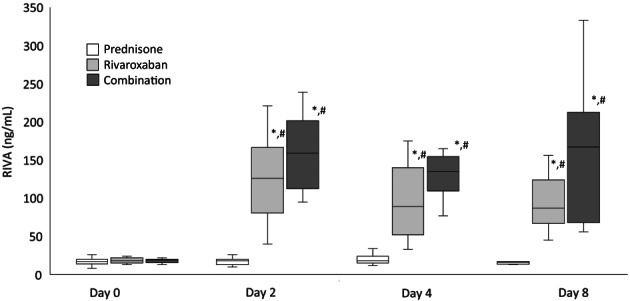
RIVA values from 9 dogs treated with prednisone, rivaroxaban, and prednisone/rivaroxaban for 8 days. The box and whiskers plot demonstrate the median (line), interquartile range (box), and total range (whiskers). *Indicates a significant (*P* < .05) difference from day 0 within a treatment group; ^#^Indicates a significant (*P* < .05) difference from the prednisone group on the corresponding day

The RIVA results for days 0, 2, 4, and 8 are presented in Figure 2. In the rivaroxaban group, there were significant increases in RIVA results from day 0 to 2 (*P* < .001, 18 [13‐33] to 126 [40‐221], median difference 108 ng/mL [95% CI: 39.1‐106.7]), day 0 to 4 (*P* < .001, 18 [13‐33] to 89 [33‐287] median difference 71 ng/mL [95% CI: 27.7‐97.9]) and day 0 to 8 (*P* < .001, 18 [13‐33] to 87 [45‐156] median difference 69 ng/mL [95% CI: 33.8‐79.8]).

In the prednisone/rivaroxaban group, there were significant increases in RIVA results from day 0 to 2 (*P* < .001, 19 [13‐22] to 159 [95‐239], median difference 140 ng/mL [95% CI: 48.7‐128.7]), day 0 to 4 (*P* < .001, 19 [13‐22] to 135 [77‐543] median difference 116 ng/mL [95% CI: 33‐157.7]) and day 0 to 8 (*P* < .001, 19 [13‐22] to 167 [56‐333] median difference 148 ng/mL [95% CI: 39.4‐133.5]).

On days 2, 4, and 8, there were no differences in the PT and RIVA values between the rivaroxaban and the prednisone/rivaroxaban groups.

### 3.2 Viscoelastometry

**FIGURE 3:**
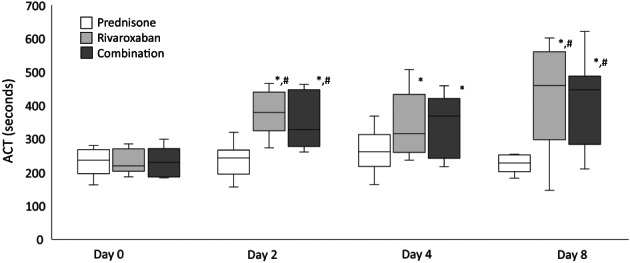
ACT values from 9 dogs treated with prednisone, rivaroxaban, and prednisone/rivaroxaban for 8 days. The box and whiskers plot demonstrate the median (line), interquartile range (box), and total range (whiskers). *Indicates a significant (*P* < .05) difference from day 0 within a treatment group; ^#^Indicates a significant (*P* < .05) difference from the prednisone group on the corresponding day

There was no difference in ACT and CR values among the before treatment groups. The ACT results for days 0, 2, 4, and 8 are represented in Figure 3. In the rivaroxaban group, there were significant increases in ACT values from day 0 to 2 (*P* < .001, 220 [187‐285.5] to 379.5 [274‐466], median difference 159.5 seconds [95% CI: 259.3‐350.5]), day 0 to 4 (*P* < .001, 220 [187‐285.5] to 316 [237‐507.5] median difference 96 seconds [95% CI: 240.9‐330]) and day 0 to 8 (*P* < .001, 220 [187‐285.5] to 460 [146.5‐602] median difference 240 seconds [95% CI: 251.9‐396]). There was a significant increase in ACT values from day 4 to 8 (P =.018, 316 [237‐507.5] to 460 [146.5‐602] median difference 144 seconds [95% CI: 313.3‐443.5]).

In the prednisone/rivaroxaban group, there were significant increases in ACT values from day 0 to 2 (*P* < .001, 230.5 [261.5‐463.5] to 328.5 [261.5‐463.5], median difference 98 second [95% CI: 248.2‐340.1]), day 0 to 4 (*P* < .001, 230.5 [261.5‐463.5] to 369 [217‐459] median difference 138.5 seconds [95% CI: 241.9‐333]) and day 0 to 8 (*P* < .001, 230.5 [261.5‐463.5] to 447 [210.5‐621.5] median difference 216.5 seconds [95% CI: 254.9‐386]).

**FIGURE 4:**
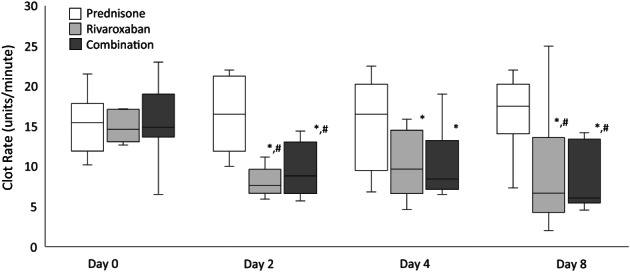
CR values from 9 dogs treated with prednisone, rivaroxaban, and prednisone/rivaroxaban for 8 days. The box and whiskers plot demonstrate the median (line), interquartile range (box), and total range (whiskers). *Indicates a significant (*P* < .05) difference from day 0 within a treatment group; ^#^Indicates a significant (*P* < .05) difference from the prednisone group on the corresponding day

The CR results for days 0, 2, 4, and 8 are represented in Figure 4. In the rivaroxaban group, there were significant decreases in CR values from day 0 to 2 (*P* < .001, 14.6 [12.6‐24] to 7.6 [5.9‐11.2], median difference 7 units/minute [95% CI: 9.4‐14.2]), day 0 to 4 (*P* = .007, 14.6 [12.6‐24] to 9.65 [4.6‐15.85] median difference 4.9 units/minute [95% CI: 10.8‐15.4]) and day 0 to 8 (*P* = .002, 14.6 [12.6‐24] to 6.6 [2‐25] median difference 7.9 units/minute [95% CI: 9.4‐15.7]).

In the prednisone/rivaroxaban group, there were significant decreases in CR values from day 0 to 2 (*P* < .001, 14.9 [6.5‐23] to 8.8 [5.7‐14.4], median difference 6 units/minute [95% CI: 10.1‐15.2]), Day 0 to 4 (*P* = .005, 14.9 [6.5‐23] to 8.4 [6.5‐19] median difference 6.5 units/minute [95% CI: 10.5‐15.6]) and Day 0 to 8 (*P* < .001, 14.9 [6.5‐23] to 6.1 [4.5‐14.2] median difference 8.8 units/minute [95% CI: 9.2‐14.9]).

On days 2, 4, and 8, there were no differences in the ACT and CR values between the rivaroxaban and the prednisone/rivaroxaban groups.

### 3.3 Correlation

**FIGURE 5:**
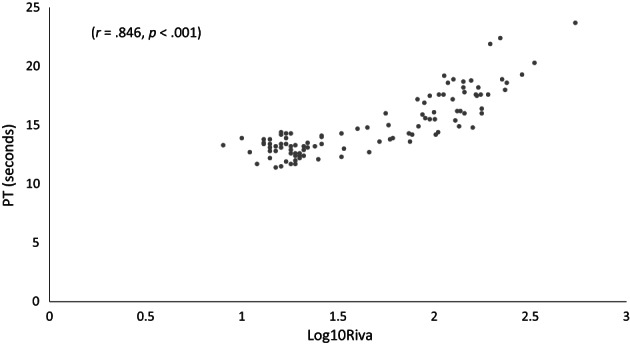
Scatterplot showing the relationship between log10Riva (rivaroxaban‐specific anti‐Xa activity [RIVA]) and PT (seconds) for 9 dogs treated with prednisone, rivaroxaban, and prednisone/rivaroxaban for 8 days. The correlation (*r*) and the associated significance (*P*‐value) are displayed at the top of the graph

Pearson's correlation (*r*) and significance (*P*) were assessed for all hemostasis tests. With days 2, 4, and 8 combined, there was a strong correlation between RIVA and PT results (*r* = .846, *P* < .001). There was a moderate correlation between RIVA and ACT (*r* = .615, *P* < .001), and RIVA and CR (*r* = −.638, *P* < .001). The correlation between RIVA and PT became stronger during drug administration [day 2 (*r* = .810, *P* < .001), day 4 (*r* = .863, *P* < .001), and day 8 (*r* = .885, *P* < .001)]. With all days combined, there was a moderate correlation between PT and ACT results (*r* = .507, *P* < .001) and PT and CR (*r* = −.547, *P* < .001) (Figure 5 and Supplemental S1).

## 4 DISCUSSION

The present study demonstrates the anticoagulant effects of rivaroxaban are not affected by the concurrent adminstration of prednisone in healthy dogs. Further investigation into the effects of rivaroxaban and glucocorticoids on anticoagulation variables in hypercoagulable dogs with conditions such as IMHA is warranted.^6^, ^7^, ^20^

The present study demonstrates the anticoagulant effects of rivaroxaban are not affected by the concurrent administration of prednisone in healthy dogs. The RIVA assay is considered the gold standard method for monitoring rivaroxaban therapy, 14 but in our study, there was a strong correlation between the PT and RIVA assay, suggesting the PT could be a useful assay for therapeutic drug monitoring.

Rivaroxaban is an oral anticoagulant that directly inhibits coagulation FXa.^9^, ^10^, ^11^, ^21^ Because of the predictable pharmacodynamic profile of rivaroxaban, dose modification based on laboratory monitoring is not routinely performed in humans.^22^, ^23^ However, because the use of rivaroxaban as thromboprophylatic therapy in dogs is relatively new and because the pharmacodynamics of the medication in hypercoagulable dogs are not fully understood, it is currently recommended that clinicians monitor the anticoagulant intensity of dogs receiving rivaroxaban and perform dose adjustments as necessary.^14^

The mechanism of exogenous glucocorticoid‐induced hypercoagulability is not completely established. Several proposed mechanisms are associated with alterations in secondary hemostasis, including decreased fibrinolysis, increased fibrinogen concentration, and decreased antithrombin activity.^1^, ^2^ In healthy dogs, an immunosuppressive dosage of prednisone caused an increase in mean global clot strength and maximum amplitude based on thromboelastography.^2^ These findings differ from the results of our study, which did not demonstrate an increase in hypercoagulability as measured by viscoelastometry in healthy dogs receiving prednisone alone. One potential explanation for this difference is that our study used a different instrument to assess coagulation. The global assessment of coagulation used in this study was based on viscoelastometry, utilizing a Sonoclot analyzer. Similar to thromboelastography, the Sonoclot measures the entire coagulation process by detecting changes in viscosity during clot formation. The Sonoclot has been established in human medicine as a bedside instrument for monitoring the anticoagulant effects of heparin.^24^ Typically, since both are dynamic measures of change in sample viscosity during clotting, thromboelastrography and viscoelastometry would be expected to yield comparable results, but differences between techniques (eg, instrumentation, data collection and derived variables) could explain the discrepant results between our study and the prior study by Flint and others.^2^

Although rivaroxaban‐calibrated anti‐Xa activity assays are considered the gold standard bioassays for monitoring rivaroxaban therapy,^14^, ^25^, ^26^ these diagnostic tests are not routinely available for immediate therapeutic adjustments. Additionally, the target therapeutic range of anti‐Xa activity associated with effective thromboprophylaxis for dogs has not been well‐established. The proposed therapeutic range in dogs has been extrapolated from human pharmacokinetic and pharmacodynamic studies.^27^, ^28^ Recently, PT, along with other assays of coagulation, was used to monitor anticoagulation during rivaroxaban administration in dogs.^14^, ^25^ Of the coagulation assays that were evaluated (thromboelastrography, PT, aPTT, and thrombin generation) the PT had a very strong (*r* = .915, *P* < .0001)^14^ or strong (*r* = .82, *P* < .0001)^25^ correlation with the RIVA assay. The correlation between the PT and RIVA results in our study was also strong (*r* = .846, *P* < .001), and comparable with these previous studies. Therefore, the PT has the potential to be an alternative to anti‐Xa monitoring in dogs treated with rivaroxaban. This is the first study to evaluate the ability of the SonoClot to detect the anticoagulant effects of rivaroxaban in healthy dogs. In contrast to the PT, there was an only a moderate correlation with RIVA results for both the ACT and CR. Although the SonoClot appears to have value as a point‐of‐care method for assessing the anticoagulant effects of rivaroxaban, PT assays might provide a more accurate assessment of rivaroxaban‐associated anticoagulation.

In healthy dogs, clopidogrel not only counteracts glucocorticoid‐induced platelet reactivity, but that the combination of prednisone and clopidogrel enhances platelet dysfunction.^29^ Although clopidogrel was shown to counteract glucocorticoid‐induced hypercoagulability based on assessment of primary hemostasis,^29^ this prior study did not focus on the mechanisms of glucocorticoid‐induced hypercoagulability that could impact the efficacy of drugs that affect secondary hemostasis, such as rivaroxaban. The results of our current study suggest that the combination of rivaroxaban and prednisone does not significantly alter the overall anticoagulant effects of rivaroxaban.

Our study had several limitations. First, the dogs used in this study were deemed healthy, and there was no evidence of disease or hypercoagulability. Dogs that are hypercoagulable might respond differently to prednisone and rivaroxaban. Second, our study only evaluated the coagulation status of the dogs during the first week of drug administration. It is possible that a longer period of drug administration would have provided additional assessment of long‐term drug‐induced anticoagulation. Third, our study only used one dosage of rivaroxaban. Current dosage recommendations suggest a dose of 1 to 2 mg/kg, PO, q24hours^6^; however, higher dosages, including treatment every 12 hours have been used in dogs.^26^ The dosage for this paper was selected because it was within the recommended dosage range, and too high of a dosage could potentially mask any glucocorticoid‐induced hypercoagulability, while too low of a dose might not accurately reflect a clinical scenario. Finally, our study only used a single thromboprophylactic agent. Depending on dog condition and clinician preference, multiple thromboprophylactic agents could be administered concurrently in some dogs. The use of multiple agents might allow for lower dosages of rivaroxaban while still conferring a decreased risk of thrombosis.

## CONFLICT OF INTEREST DECLARATION

Marjory Brooks is the Director of the Comparative Coagulation Section of the Diagnostic Laboratory at the Cornell University College of Veterinary Medicine. No other authors have a conflict of interest.

## OFF‐LABEL ANTIMICROBIAL DECLARATION

Authors declare no off‐label use of antimicrobials.

## INSTITUTIONAL ANIMAL CARE AND USE COMMITTEE (IACUC) OR OTHER APPROVAL DECLARATION

Approved by the Mississippi State University College of Veterinary Medicine IACUC (# 20‐314).

## HUMAN ETHICS APPROVAL DECLARATION

Authors declare human ethics approval was not needed for this study.

## Supporting information


**Supplemental S1**. Scatterplot showing the relationship between RIVA (ng/mL; rivaroxaban‐specific anti‐Xa activity [RIVA]) and PT (seconds) for 9 dogs treated with prednisone, rivaroxaban, and prednisone/rivaroxaban for 8 days
