# Supplementary figures and images for: Anticoagulant effects of rivaroxaban, prednisone, alone and in combination, in healthy dogs
Source: J Vet Intern Med. 2022 Nov 18;36(6):2009–15. doi: 10.1111/jvim.16572 (PMC9708426; doi:10.1111/jvim.16572)

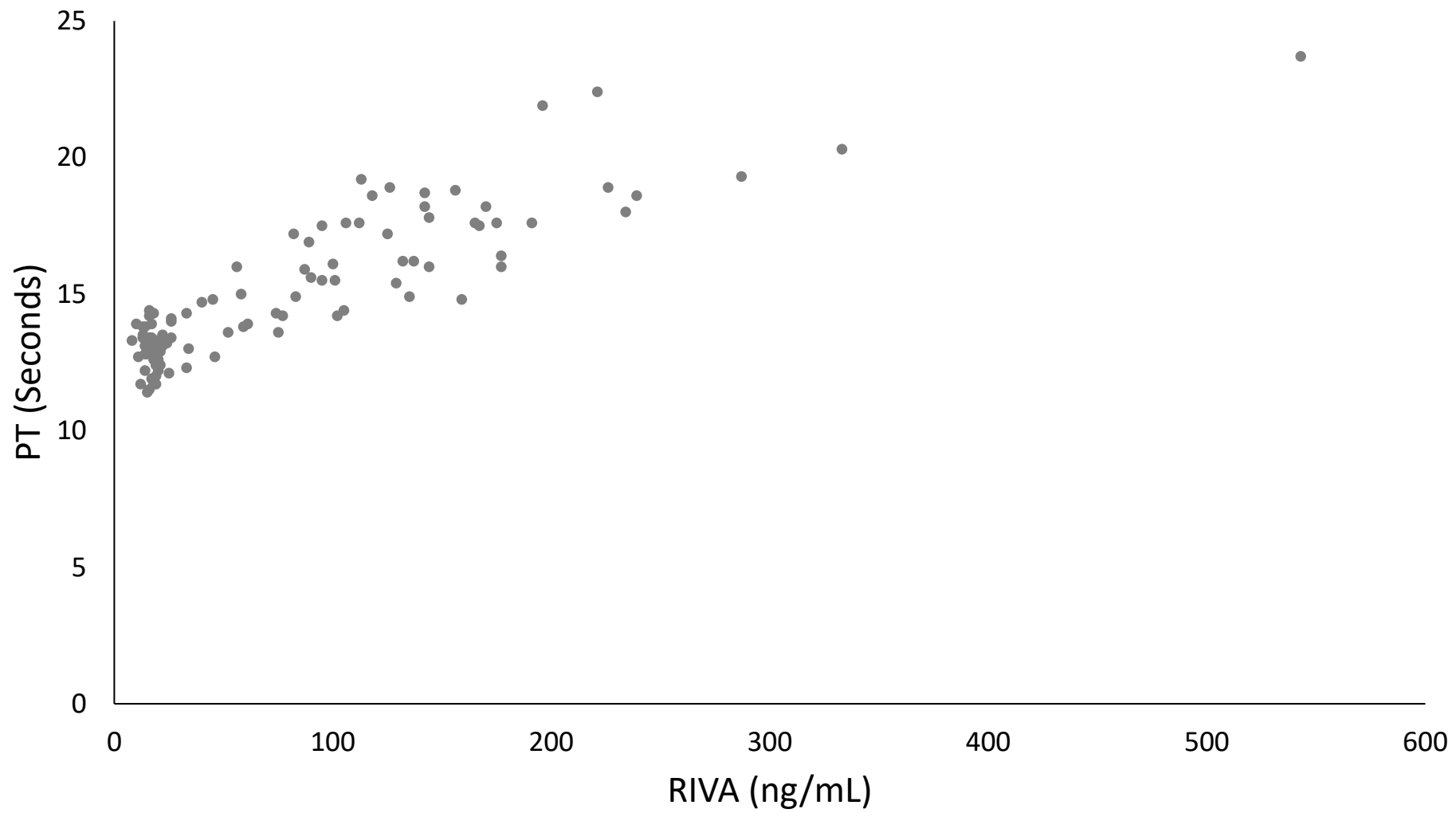

Supplement: Supplementary file 1 — Supplemental S1. Scatterplot showing the relationship between RIVA (ng/mL; rivaroxaban‐specific anti‐Xa activity [RIVA]) and PT (seconds) for 9 dogs treated with prednisone, rivaroxaban, and prednisone/rivaroxaban for 8 days [file JVIM-36-2009-s001.pdf]
